# SARS-CoV-2 Pandemic: A Comparison Between the Epidemiological Situation in Greece and Romania

**DOI:** 10.7759/cureus.54460

**Published:** 2024-02-19

**Authors:** Anastasia Rigatou, Madalina Camelia Sultana

**Affiliations:** 1 Virology, "St. S. Nicolau" Institute of Virology, Carol Davila University of Medicine and Pharmacy, Bucharest, ROU

**Keywords:** infectious diseases, balkan peninsula, virology, epidemiology, sars-cov-2

## Abstract

Introduction

Since the onset of the SARS-CoV-2 pandemic, there seems to be scarce data targeting the comparison of epidemiological data among different countries. In an attempt to reveal and characterize the epidemiological profile in the Balkan peninsula, a cross-sectional study has been conducted, aiming to retrospectively collect all the existing information regarding the SARS-CoV-2 pandemic over a period of three years, starting from March 2020 until March 2023. The comparative analysis of the epidemiological features and the main indicators between Romania and Greece can generate a good overview of the factors that can influence public health and create an adequate system of measures to limit the COVID-19 pandemic in the area. A retrospective comparative study aiming to detect and associate the main indicators determining the evolution of the SARS-CoV-2 pandemic data with the control measures adopted in Romania and Greece was performed.

Methods

Publicly available data were obtained from official sources such as the World Health Organization, the European Centre for Disease Control, the Romanian and Greek Ministries of Health, and the Romanian National Centre for Surveillance and Control of Communicable Diseases. The reported number of cases, in total and in conjunction with the age distribution, total number of deaths, and vaccination coverage, from the onset of the pandemic in March 2020 until March 2023, were collected. All officially reported cases of COVID-19 were included in this analysis. Reports with missing or incomplete values regarding the timeframe, age distribution, and vaccination status were excluded.

Results

During the timeframe of the study, from March 2020 until March 2023, Greece reported a higher number of confirmed SARS-CoV-2 cases as compared to Romania (5,910,103 cases and 3,352,356 cases, respectively). Still, in terms of the overall death toll, Romania recorded a higher mortality rate than Greece during the pandemic (67.773 deaths *vs.* 36.372 deaths).

Concerning both cumulative incidence rates and the 14-day case notification rate per 100.000 inhabitants, it is evident that Romania exhibited greater numbers throughout the course of the pandemic. Although it is not clearly stated, the compulsory vaccination of elderly people that was set as a high priority in Greece may have contributed to the above results.

In terms of the 14-day death notification rate per 100.000 inhabitants in 2020 and 2021, Romania showed a higher rate than Greece, while Greece reported a greater rate in 2022 and up until March 2023.

Between 2020 and 2023, Greece presented both a higher number of vaccinated individuals and a higher vaccination coverage with two doses (7,034,695 individuals, 70% of the general population), as compared to Romania (6,467,804 individuals, 33.68% of the general population, p<0.0001).

Conclusions

Despite the similar restrictions and preventive actions adopted by Romania and Greece, some of the epidemiological data between the two countries tends to differ. It must not be ignored that every nation should be considered a unique entity with distinct features, including individuals, customs, and policies, rather than being categorized with other countries based on geographic proximity or regionalization.

## Introduction

Since the onset of the SARS-CoV-2 pandemic, the virus has spread and afflicted the global population. In isolated cases in Wuhan, China, due to its high-risk transmission rate, SARS-CoV-2 was deemed the most lethal among the coronaviruses [1]. Over 67 million cases and over six million deaths were reported due to SARS-CoV-2 as of January 31, 2023 [2]. All initial research efforts focused on the clinical progression and characteristics of the virus, with the aim of developing advanced therapeutic strategies. Governments and scientists throughout the globe recognized the urgent need for enhanced public health precautions in order to fully comprehend the epidemiology of the novel virus and define its potential implications based on prior outbreaks such as Middle East respiratory syndrome (MERS) and influenza [3].

As only a portion of all acute infections were diagnosed and documented, the reported cases significantly underestimated the overall impact of COVID-19 [4]. Even if the pandemic is gradually completing its emergency phase, continuous viral circulation and the appearance of novel subvariants cause new surges to happen in Europe. Unlike the influenza virus or other seasonal respiratory diseases, new infections can continuously emerge. A significant decline in diagnostic testing across the continent makes it challenging to evaluate the scope of the most recent COVID-19 waves [5-7]. Various findings across the USA and Europe have exhibited that after adjusting for variables, the rate of previous exposure to SARS-CoV-2, which was characterized by seropositivity, surpassed the incidence of reported cases by at least 10 times [4,8,9]. Numerous studies have attempted to decipher the epidemiological profile and features of the virus. Nonetheless, there seems to be a lack of literature and a considerably limited number of studies aiming to compare large epidemiological data among different countries [10].

Therefore, the aim of the current cross-sectional study is to gather all the existing information about the SARS-CoV-2 pandemic and immunization systems from Romania and Greece retrospectively. This study also aims to compare the epidemiological status of the two countries as well as to distinguish the epidemiological and immunization profiles in the Balkan region.

The objectives of the study are to generate recommendations for enhancing the epidemiological response to the current SARS-CoV-2 pandemic, to enhance preparedness for a possible new epidemic event, and to provide governments with the required resources to manage the SARS-CoV-2 negative outcomes.

## Materials and methods

We conducted a cross-sectional study to collect all the existing information regarding the SARS-CoV-2 pandemic over a period of three years, from March 2020 up until March 2023. A retrospective comparative study aiming to detect and associate the main statistical indicators defining the evolution of the COVID-19 pandemic data with the control measures adopted in Romania and Greece was performed.

Publicly available data were obtained from official sources, such as the World Health Organization website, the European Centre for Disease Control websites, the Romanian and Greek Ministry of Health, and the Romanian National Centre for Surveillance and Control of Communicable Diseases [11-14]. The total reported cases, in conjunction with the age distribution, total number of deaths, and vaccination coverage from the onset of the pandemic until March 2023, were collected. All officially reported cases of COVID-19 were included in this analysis. Reports with missing or incomplete values regarding the timeframe, age distribution, and vaccination status were excluded. 

Statistical analysis

A descriptive statistical analysis was used; categorical variables were summarized as frequencies and percentages; continuous variables, according to their distribution, as means with standard deviation or medians with the first and third quartiles (interquartile range; IQR). The Microsoft Excel 365 program was used to collect and analyze data. Weekly cumulative cases and deaths were used to describe the time trend. The statistical analysis was conducted with GraphPad Prism (version Prism Mac 7.0a), using the Pearson correlation test for direct correlation of the quantitative variables and the t-test to compare means for the variables in different groups; p-values below 0.05 were considered statistically significant. 

## Results

General comparison of SARS-CoV-2 epidemic figures in Romania and Greece

**Figure 1:**
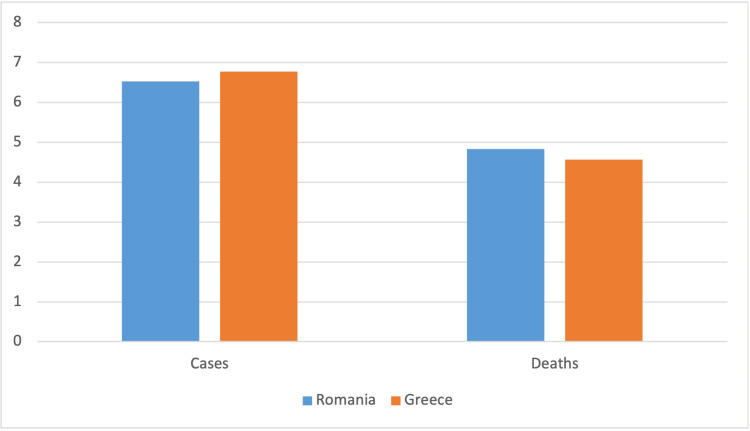
Total number of confirmed cases and deaths in Greece and Romania y-axis in log-10 scale

The outbreak of the COVID-19 pandemic in Romania and Greece was declared on the same day, namely February 26th, 2020. During the current study, the two countries reported 9,262,459 confirmed cases. Although Romania has a bigger population than Greece (18,889,702 population vs. 10,283,270 population), Greece reported a higher number of confirmed SARS-CoV-2 infection cases (5,910,103 cases and 3,352,356 cases, respectively). Still, in terms of the overall death toll, Romania recorded a higher number of mortal cases than Greece (67,773 deaths vs. 36,372 deaths) (Figure 1).

**Figure 2:**
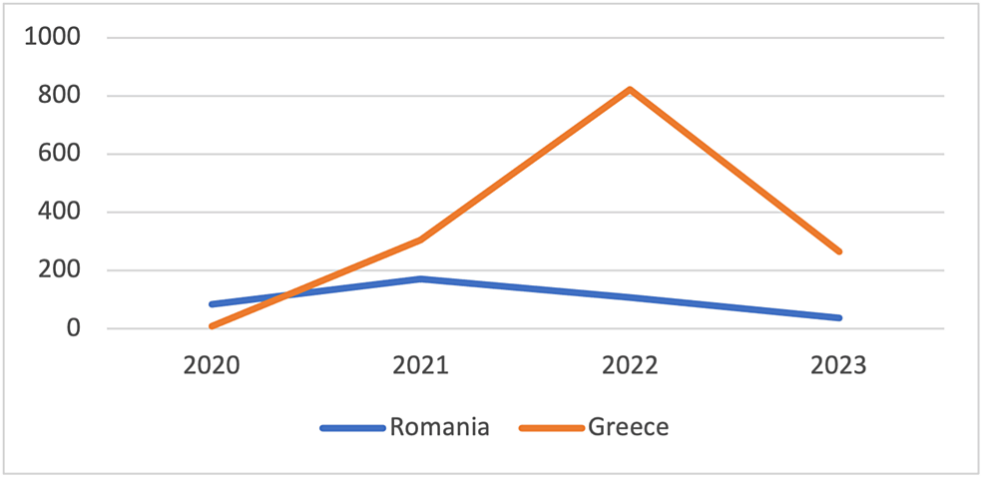
14-day case notification rate per 100,000 inhabitants per year

During 2020, the Romanian median 14-day notification rate per 100,000 inhabitants was 84.2 (IQR: 20.53-249.19), in comparison to Greece, which exhibited a median 14-day case notification rate of 10.04 (IQR: 3.81-54.68); p<0.0001. In the next three years, Greece reported a higher 14-day notification rate (2021: median: 304.71 [IQR: 184.42-397.23], 2022: median: 829.23 [IQR: 657.32-189.06], and 2023: median: 264.76 [IQR: 256.67-552.23]) than Romania (2021: median: 170.82 [IQR: 42.69-290.18], 2022: median: 107.52 [IQR: 34.47-313.28], and 2023: median: 36.49 [IQR: 29.7-36.9]) (Figure 2).

**Figure 3:**
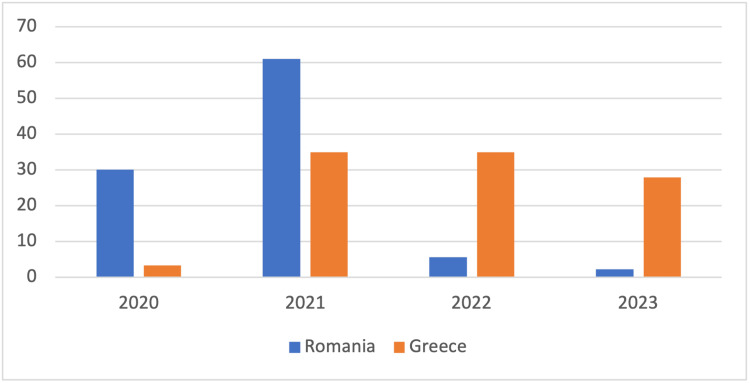
14-day death notification rate per 100,000 inhabitants per year

Regarding the 14-day death notification rate per 100,000 inhabitants in 2020 and 2021, Romania (2020-median: 30.02 [IQR: 14.24-61.3] and 2021-median: 61.06 [IQR: 19.76-107.37]) presented a higher rate compared to Greece (2020-median: 3.37 [IQR: 1.22-10.86] and 2021-median: 46.64 [IQR: 32.92-82.64]), while in 2022 and until March 2023, Greece (2022-median: 34.89 [IQR: 26.5-71.54] and 2023-median: 27.9 [IQR: 19.76-33.62]) reported a higher rate than Romania (2022-median: 5.65 [IQR: 1.9-16.07] and 2023-median: 2.19 [IQR: 2.03-2.5]) (Figure 3).

Concerning death rates, even though Romania reported a higher number of deaths in 2020 and 2021 (18.456 deaths in 2020 and 40.515 deaths in 2021), Greece recorded a higher number of deaths in 2022 and 2023 (13.625 deaths in 2022 and 1.240 deaths until March 2023). 

Vaccinations in Romania and Greece 

Both countries started their vaccination campaigns on the same day, December 27^th^, 2020. The Romanian government developed a system of prioritization for the administration of COVID-19 vaccines, with healthcare personnel being vaccinated first, then the elderly living in nursing homes and other institutional settings, those over 65, and patients with preexisting medical issues. Individuals in high-risk professions were the next priority group, followed by the majority of the population. On the other hand, the Greek government established an online platform that provided individuals, according to their age group, with a designated time frame during which they could start getting vaccinated. For people under the age of 29, the platform opened in June 2020, and thus, a considerable number of young people who wanted to get vaccinated could not be immunized.

Both states used Pfizer, Johnson & Johnson, and Pfizer Pediatric (5-11 years) vaccines. Romania employed the AstraZeneca and Moderna vaccines, whereas Greece additionally used Novanax, Pfizer 6M-4E, and Pfizer BA45. Both Greece and Romania administered the Pfizer vaccine to children aged 12 to 17 years old, and both nations used the Pfizer Pediatric vaccine for children aged 5 to 11. Moreover, Greece provided the Pfizer 6M-4E vaccination to children between the ages of 6 months and 4 years. 

While Romania reported a higher number of people vaccinated with one dose (8,186,546 citizens) compared to Greece (7,923,638 citizens), Greece presented a higher percentage of vaccinated people in the total population than Romania (75.46% vs. 42.55%); p<0.0001. Furthermore, regarding the number of people vaccinated with two doses, Greece presents both a higher number and a higher vaccination percentage of the total population (7,034,695 citizens were vaccinated with the second dose, 70% of the general population) as compared to Romania (6,467,804 citizens were vaccinated with the second dose, which is 33.68% of the general population; p<0.0001).

**Figure 4:**
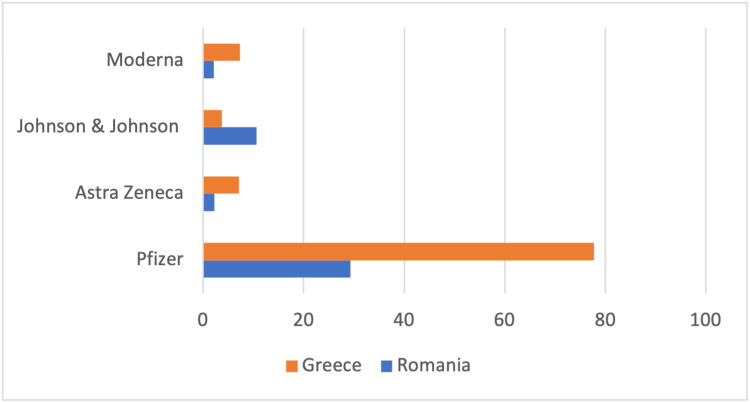
Percentages of people vaccinated with each type of vaccine

Comparing the percentages of people vaccinated with each type of vaccine that both countries administered, it can be concluded that Greece has a higher percentage of usage of each type of vaccine (Pfizer [77.74%], AstraZeneca [7.07%], Johnson & Johnson [3.75%], and Moderna [7.34%]) as compared to Romania (Pfizer [29.28%], AstraZeneca [2.26%], Johnson & Johnson [10.6%], and Moderna [2.16%]) (Figure 4).

It is worth mentioning also that Greece recorded a higher percentage of vaccination across all age groups (Table 1), with important differences seen in all age groups (Table 1).

**Table 1:** The comparison of the vaccinated population, distributed by age groups

Age groups	Number (%) of vaccinated population Romania	Number (%) of vaccinated population Greece
<18	259315 (1,35%)	441464 (4,13%)
18-24	454325 (2,37%)	448219 (4,2%)
25-49	2331054 (12,14%)	2366929 (22,17%)
50-59	1082540 (5,69%)	1192205 (11,17%)
60-69	1109741 (5,78%)	1140480 (10,68%)
70-79	650908 (3,39%)	900773 (8,43%)
80+	216231 (1,13%)	655000 (6,13%)

**Figure 5:**
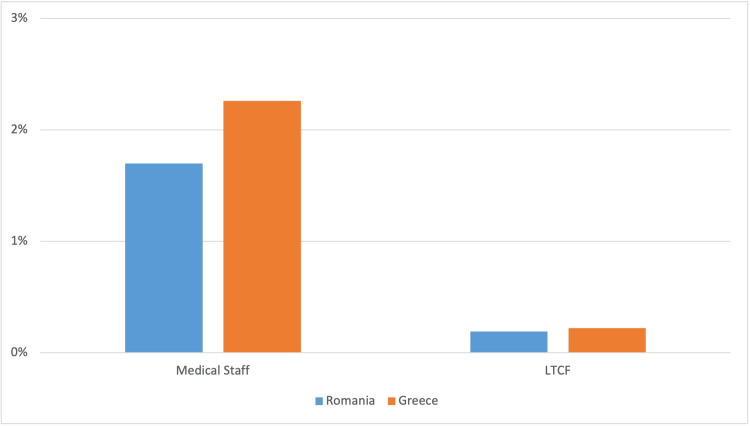
Percentage of vaccinated medical staff and LTCF LTCF: long-term care facilities

Moreover, the fact that Greece exhibited a higher number of vaccinated medical personnel (2.26% vs. 1.7%; p=0.002), including those working in long-term care facilities (LTCF: 0.22% vs. 0.19% p= 0.639), as compared with Romania, should also be mentioned (Figure 5).

The period between vaccine doses also varies between the two countries. For the Pfizer and Moderna vaccines, Greece advised an eight-week recommended time interval between the first and second doses, while in Romania, the recommended interval was 21 days for the Pfizer vaccine and 28 days for the Moderna vaccine. While in Greece, vaccination was mandatory for citizens over 60 years of age, facing fines if they were not vaccinated, in Romania, vaccination was optional. Both countries implemented free vaccine administration.

Both Greece and Romania employed similar therapeutic approaches aligned with the respective national protocols and guidelines. According to these protocols, the treatment of COVID-19 depends on the severity of the infection. Hence, no medical intervention was recommended for the asymptomatic cases. On the other hand, non-steroid anti-inflammatory drugs are advisable for mild forms of the disease, while a variety of medications are administered for severe and chronic cases.

Finally, similar restrictions and preventive actions were adopted by Romania and Greece, including lockdowns, emergency states, limiting business activities, the closure of schools and facilities, social distancing, travel bans, and the mandatory wearing of masks.

## Discussion

The most significant counterparts and differences between Romania’s and Greece’s responses to the SARS-CoV-2 pandemic and programs of vaccination are highlighted in this research. 

The footprint of the pandemic on the European continent was diverse, with significant variations on the timelines and peaks of the outbreak across the region. In the setting of 2020, when the virus was already on Europe´s doorstep, in contrast to other countries mostly located in the southern and western regions, such as Italy, the Balkan peninsula started encountering COVID-19 patients in February 2020. Particularly, both Romania and Greece recorded their first cases on February 26th [15].

As in every other European state, Romania and Greece were under the guidance and surveillance of the European Parliament, which supervised and coordinated the countermeasures against COVID-19, providing the necessary funds and means in order to hold back the spread of the virus [16]. In addition, the countries´ governments, following the recommendations and the direction paved by the World Health Organization, implemented supplementary policies similar to those observed in other countries in an attempt to mitigate, at times, the ongoing situation. The concomitantly prompt centralized and individual strategies led to a decelerating increase in infected and deceased records compared to Western Europe, a situation that was effectively preserved until the end of spring [17]. As a result, and despite the imposing of harsh restrictions, such policies met an initially substantial level of public trust and compliance, reflecting the fruitful and constructive coordination between the authorities and the citizens [17].

As the summer of 2020 approached, the limited numbers of documented cases and deaths led the restrictions to be lifted in a rather unbalanced decision, which was mainly influenced by political and economic reasons. The infection rates were rising rapidly and progressively, leading to the implementation of even stricter measures. As a result, the credibility of governments in the eyes of the public was fading [17]. However, the marching of the second wave towards Romania and Greece could not be intercepted, with tremendous effects and numerous bereavements [15].

During that period, the two countries confronted various difficulties, mainly due to the fact that numerous tourists were traveling across the countries, reflecting the still-unaddressed drawbacks of the inadequate healthcare system and the inefficacy of the central administration [16].

Recent studies stand for the investigation and examination of the COVID-19 epidemiological profile across borders. Certainly, comparable characteristics were observed across adjacent nations, establishing a “cross-border effect” model about the morbidity and mortality rates throughout the pandemic's timeline [18]. Supplementary, such models will also facilitate the evaluation of the counteracting measures; the ability of one country to contain and immunize against COVID-19 is mirrored in the neighboring countries and vice versa [19]. To maintain low infection and mortality numbers, as well as to alleviate the healthcare system, it is vital that countries re-adhere to a central European commitment instead of “enforcing” individual policies [19]. Data comparing the epidemiological profile across Balkan countries, including Romania, are available; however, there is a dearth of literature when it comes to the comparison between Romania and Greece. Hence, our work is the first study reporting on such epidemics. Overall, although Romania has a larger population, Greece has reported a higher number of confirmed cases of COVID-19 as compared to Romania (5,910,103 cases and 3,352,356 cases, respectively). Still, in terms of the general mortality figures, Romania has recorded an increased number of deaths than Greece during the pandemic (67.773 deaths versus 36.372 deaths) until March 2023.

According to a comparative study, Romania's mortality rates exceeded the European average, which may be attributed to various factors, of which the inability of the healthcare system to tackle severe cases, the patients´ comorbidities, the limited number of daily tests, and the geographical distribution of the cases are of primary importance [16]. On the contrary, in Serbia, which can be deemed equivalent to Romania in terms of its social dynamic, the epidemiological situation was quite dissimilar. The Serbian government made the daily conduct of high-volume COVID-19 diagnostic tests one of the top priorities in confronting the pandemic. Such measures resulted in an elevated incidence rate, which reflects the on-time detection and management of the majority of the cases, leading to constantly below-average European mortality rates [16,20]. Based on our research, we found out that although Romania has a larger population than Greece, the latter has reported a higher number of confirmed cases as compared to Romania. But still, in terms of the overall death toll, Romania has recorded an increased number than Greece during the pandemic, which can be partially attributed to the different number of tests conducted. Supplementary, Hungary, one of the first countries to implement lockdowns, managed to maintain low infection and death rates by successfully identifying and quarantining individual subjects [15].

Based on a paper published in 2020 that investigated the cumulative excess mortality among different countries in Europe, a classification system according to the mortality dynamics throughout the pandemic may be applicable in these states. In more detail, multiple countries like the United Kingdom, France, Spain, Italy, Belgium, and Sweden recorded a constantly increasing mortality toll during the first wave (spring 2020), which persisted in the second wave, with the excess mortality rate surpassing 10% [15].

On the other hand, countries located in the central-east region, along with countries in the Balkan peninsula, e.g., Romania, Slovenia, Hungary, Bulgaria, and Greece, exhibited low mortality rates at the beginning of the pandemic; however, the cumulative excess mortality dramatically increased (>10%) during the second wave, while Scandinavian and Baltic states reported a cumulative excess mortality of 7% [15]. Our research showed that, despite Romania reporting a larger number of deaths in 2020 and 2021, Greece recorded a higher number of deaths in 2022 and 2023. 

The post-socialist character of the Romanian community, where its people are used to discipline and follow the regulations, and concomitantly, the inadequate infrastructure of the Greek health-care system, which was not reenforced properly during the pandemic, may take partial responsibility for these results [15].

Concerning both cumulative incidence rates and the 14-day case notification rate per 100,000 inhabitants, it is evident that Romania exhibited greater numbers throughout the course of the pandemic. Although not clearly stated, the compulsory vaccination of elderly people that was set as a high priority in Greece may have contributed to the above results. Moreover, according to studies, the lower the temperature and humidity, the more cases occur. COVID-19 cases rose at temperatures ranging from 0 to 17°C. These parameters may also partially account for the increased 14-day case notification rate that Romania presented compared to Greece [21-23]. In terms of the 14-day death notification rate per 100,000 inhabitants in 2020 and 2021, Romania showed a higher rate than Greece, while Greece reported a greater rate in 2022 and up until March 2023. The reason for the difference is not entirely obvious, but several factors can be linked and further tested, including co-morbidity rates, individual factors, vaccination rates, a lack of protective measures, etc.

During the 4th quartile of 2020, the newly developed vaccines started getting implemented in the fight against the virus, and the treatment armamentarium was reinforced substantially. Both Romania and Greece, through the EU´s “Joint Procurement Agreement,” were able to secure enough vaccines to initiate an anti-COVID-19 vaccination campaign and combat the spread of the virus [24].

Both countries started their vaccination campaigns in December 2020, but with different approaches: the Romanian government developed a system of prioritization for the administration of COVID-19 vaccines, with healthcare personnel being vaccinated first, followed by the risk groups. The Greek government established a specific platform that gave each age group a designated time frame during which they could start getting vaccinated. Truly, a longitudinal observational study comparing data among 35 European countries highlighted the fact that the more individuals getting fully vaccinated, the more the case fatality rates decline. In addition, the same authors note that some Balkan countries with lower vaccination rates, like Romania and Bulgaria, have greater infectivity rates compared to other nations [18]. Keeping in mind that, as countries had already been dealing with the pandemic for over a year, the preservation, maintenance, and successful completion of the vaccination campaign faced many obstacles, including but now limited to the emergence of new variants, the “marks” of these obstacles are reflected in the epidemiological data of each nation [25].

Regarding the two countries of this study, it is shown that Greece presented a higher percentage of vaccinated people, both with one dose (75.46% versus 42.55%; p<0.0001) and furthermore with the second dose (70% versus 33.68%; p<0.0001) as compared to Romania, probably being one of the explanations for the higher number of deaths recorded in Romania.

In the first semester of 2021, the data coming from Romania was encouraging since almost 20% of the population was fully vaccinated, resembling the overall European average of the until then completely immunized people. From mid-summer 2021 on, the progress of the campaign seemed to decline drastically; nearly 27% of the total population was vaccinated, and, in particular, only 35% of the people over 60 years of age were immunized, compared to the 83% of the European average. At the end of the same year, merely 40% of the population was vaccinated, while for those over 60 years of age, the vaccination rates were below 50%, significantly lower than the European average, which was 70% and 90%, respectively [25]. Greece, on the other hand, despite the fact that the platform opening date for people under the age of 29 was delayed (June 2021), affecting the entire age cohort, which should have been vaccinated earlier, exhibited better results in the immunization of the elderly. It is worth mentioning that in Greece, the vaccination was mandatory for citizens over 60 years of age, including fines if not obeying, while in Romania, the immunization was totally voluntary.

The not-so-successful Romanian vaccination program mirrors the already-known general mistrust of vaccines; 25 years ago, the abortive attempts of HPV vaccination initiatives came as the consequence of vaccine hesitancy [16]. Supplementary, the unsuccessful control of the measles epidemic due to the rising anti-vaccination movement, along with the poor trust in the healthcare system and government work, set a negative precedent that had and still partially needs to be overcome for the adequate protection of the citizens against the virus [16,17,24]. The two aforementioned examples advocate for the common vaccine rejection beliefs, which, according to a 2021 survey on attitudes towards SARS-CoV-2 immunization conducted in 26 European countries, reached a surprising 42% of the total population: 26% were not willing to vaccinate, and 16% were neutral [26]. The situation is similar in Romania, Bulgaria, and Ukraine, with important consequences for public health [16]. When it comes to today's piecemeal lift of the COVID-19 restricting measures, the continuation of the vaccination program and the concomitantly ongoing epidemiological vigil are most likely the best ways of moving forward and dealing with a new reality [18,19].

Strengths and limitations

The present study has some strengths and limitations that should be taken into account. The week point of the present study is the time and geographic limitation, as it comprises the first three years of SARS-CoV-2 pandemics in two members of the European Union.

The strong point of the study is the fact that this is, to our knowledge, the first study to compare Romania and Greece as related to the main statistical indicators defining the evolution of the COVID-19 pandemic data and the main control measures and immunization program.

## Conclusions

Despite the similar restrictions and preventive actions adopted by Romania and Greece, some of the epidemiological data between the two countries tends to differ. Over the course of the pandemic and until the completion of the study, Greece reported a higher number of confirmed SARS-CoV-2 cases, a 14-day notification rate, and a higher percentage of vaccinated people in the total population than Romania. When it comes to the overall death toll, Romania recorded a higher number of deaths than Greece.

Considering the opportunity for cross-border effects, it could be helpful to examine the situation in a wider context, including adjacent nations. Nonetheless, it must not be ignored that every nation is a unique entity with distinct features, including individuals, customs, and policies, rather than being categorized with other countries based on geographic proximity or regionalization. Sustaining vaccination campaigns and conducting ongoing epidemiological surveillance in parallel is probably the best course of action for addressing the emerging SARS-CoV-2 pandemic. In order to achieve an efficient and unified surveillance system that is capable of rapidly adapting to potential epidemics or pandemics by ensuring flexible organizational and funding conditions and regulations, it is imperative that all EU countries reorganize their health information systems and standardize them for all levels of medical services.
